# A Study on Strategic Provisioning of Cloud Computing Services

**DOI:** 10.1155/2014/894362

**Published:** 2014-06-15

**Authors:** Md Whaiduzzaman, Mohammad Nazmul Haque, Md Rejaul Karim Chowdhury, Abdullah Gani

**Affiliations:** ^1^Center for Mobile Cloud Computing Research (C4MCCR), University of Malaya, 50603 Kuala Lumpur, Malaysia; ^2^School of Electrical Engineering and Computer Science, The University of Newcastle, Callaghan, NSW 2308, Australia; ^3^Department of Electrical & Computer Engineering, University of Western Ontario, London, ON, Canada N6A 3K7

## Abstract

Cloud computing is currently emerging as an ever-changing, growing paradigm that models “everything-as-a-service.” Virtualised physical resources, infrastructure, and applications are supplied by service provisioning in the cloud. The evolution in the adoption of cloud computing is driven by clear and distinct promising features for both cloud users and cloud providers. However, the increasing number of cloud providers and the variety of service offerings have made it difficult for the customers to choose the best services. By employing successful service provisioning, the essential services required by customers, such as agility and availability, pricing, security and trust, and user metrics can be guaranteed by service provisioning. Hence, continuous service provisioning that satisfies the user requirements is a mandatory feature for the cloud user and vitally important in cloud computing service offerings. Therefore, we aim to review the state-of-the-art service provisioning objectives, essential services, topologies, user requirements, necessary metrics, and pricing mechanisms. We synthesize and summarize different provision techniques, approaches, and models through a comprehensive literature review. A thematic taxonomy of cloud service provisioning is presented after the systematic review. Finally, future research directions and open research issues are identified.

## 1. Introduction

Cloud computing is the distributed computing model that provides computing facilities and resources to users in an on-demand, pay-as-you-go model [1]. The aim of the cloud computing model is to increase the opportunities for cloud users by accessing leased infrastructure and software applications anywhere and anytime [2]. Therefore, cloud computing offers a new type of information and services that broadens the brand new vision of information technology (IT) services. The recent publicity surrounding cloud computing and at the same time the rise of smart mobile device help us to envision mobile cloud computing (MCC) [3]. MCC is a distributed computing model combining cloud and mobile computing [4, 5]. The objective of MCC is to enhance the computing and processing power of mobile devices by offloading tasks to cloud data centres [6–8]. In the cloud, resources are hosted as software, database services, virtual servers (virtual machines), hardware, complete work flows, or complex configurations of distributed computing systems and applications for provisioning [9, 10]. These resources are provisioned as services and offered to the customer by the cloud service provider (CSP). Therefore, the CSP leverages cloud services in two forms: service and cloud provider. A cloud provider is the entity that offers and upholds a cloud and may offer internally developed services on the cloud. A service provider is an entity that crafts and preserves services for running and publishing in the cloud [11–13].

Service is a type of relation between the service provider and the service user. Therefore, service provisioning is the process of enabling the service customers to access the predefined resources and enjoy the required provisioned services [14–16]. The resources are in the forms of hardware, software, computation, and storage. Essentially, the primary interaction between the customer and service provider depends on service provisioning [17]. Thus, service provisioning plays a vital role for both the CSP and the cloud users. The CSP must provide the required and promised services, promised in the service level agreement (SLA), such as quality of services (QoS), availability, and pricing, and the services must satisfy the user requirements, such as on-demand availability, scalability, elasticity, security, and exact billing [18–20].

One of the objectives of service provisioning is fair comparison among the available services. Thus, the user can compare the different cloud service offerings according to their needs and prioritize them based on several predefined dimensions [21]. However, the services provisioned to the user should meet certain service compliances and policies. Hence, the CSP assures the client of data protection, confidentiality, and security by complying with international compliance authorities, such as the National Institute of Standards and Technology (NIST), European Network and Information Security Agency (ENISA), health insurance portability and accountability act (HIPAA), and cloud security alliance (CSA). In addition, service provisioning should maintain the QoS, SLA, and user service requirements. All of these factors enable the user to compare the provisioned services, predict them, and rank them according to the user's needs to select the best available services. Therefore, from the customer's point of view, service provisioning is a very important aspect of cloud services [22, 23].

This systematic review of cloud service provisioning enhances the knowledge for the general reader and new researchers. Hence, this review has a significant impact in this domain. Therefore, we reviewed the state-of-the-art of the art service provisioning objectives, essential services, topologies, user requirements, necessary metrics, and pricing; we synthesize and summarize different provision techniques, approaches, and models through a comprehensive literature review. A thematic taxonomy of cloud service provisioning is presented after the systematic review. Finally, future research directions and open research issues are identified.

The rest of this paper is organized as follows: Section 2 expresses the service provisioning background with definition, topology, and taxonomy.  Section 3 discusses the objectives of service provisioning.  Section 4 offers an overview of key areas of service provisioning.  Section 5 introduces the service provisioning requirements and Section 6 deals with metrics.  Section 7 focuses on several models, methods, and provisioning techniques.  Section 8 explains the future research issues and challenges. Finally, Section 9 provides the conclusion. For better understanding for the general readers, we present a list of acronyms of commonly used words throughout this paper in the Abbreviations Section.

## 2. Service Provisioning Definition, Topology, and Taxonomy

Cloud service provisioning is a manner of providing customers access to resources to complete the desired tasks required by the customer. The hardware, software, or computational tasks can be the form of provisioned resources [24]. In topological perspective, service provisioning is divided into two parts: single cloud and intercloud. A single cloud computing data center is used by the client who brings several challenges. The unavailability of cloud service can leave thousands of customers relying solely on limited essential and paid resources. Grozev and Buyya [25] introduce and present taxonomies of federated cloud architectures, mechanism of application brokering, and the current environments. Formally, intercloud computing is defined as in [26]:

“a cloud model that, for the purpose of guaranteeing service quality, such as the performance and availability of each service, allows on-demand reassignment of resources and transfer of workload through an interworking of cloud systems of different cloud providers based on coordination of each consumer's requirements for service quality providers SLA and use of standard interfaces.”

**Figure 1:**
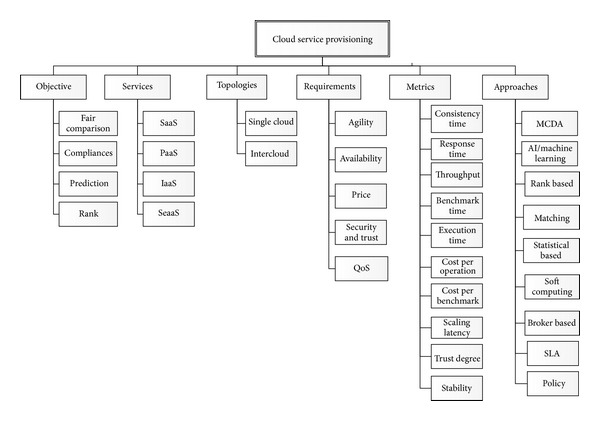
Taxonomy of cloud service provisioning.

The state-of-the-art thematic taxonomy of service provisioning is presented by classifying several vital key issues for further discussion.  Figure 1 shows the taxonomy of service provisioning selection, comprising approaches, objectives, requirements, metrics, techniques, services, and topologies. There are several approaches to service selection, including brokerage based, SLA matching, and policy based, heuristic, and holistic. The techniques involved in different service provisioning approaches include the genetic algorithm, game theory, and multicriteria decision-making. The main objectives are availability, scalability, comparison capability, and the CSP's compliance with the relevant regulations. In addition, the primary requirements include QoS, web services (WS), security aspects, pricing, and the elastic capability of the services. The essential metrics are divided into three broad aspects: storage, network, and computation. Provisioning is fundamentally based on three core service models: infrastructure as a service (IaaS), platform as a service (PaaS), and software as a service (SaaS). It may derive from a single cloud or within the multicloud via the interaction of several service providers.

By combining cloud and internet of things, Salvatore Distefano et al. envision and propose a new concept “cloud of things” (CoT) [27]. They aggregated heterogeneous resources and tailored thing-like semantics by enabling things as a service. Moreover, they model the cloud service computing as a sensing and actuation as a service (SAaaS), thus provisioning services by the sensors and actuators [28]. An architecture is designed for pervasive ICT structure generating the nearby environment data by sensors and actuators through cloud [29].

## 3. Objective of Service Provisioning 

The strategic objectives of provisioning cloud services have a paramount importance. We describe the major objectives as follows.

### 3.1. Fair Comparison

One of the objectives of service provisioning is the fair comparison among the available services or with the CSP. Generally, users compare different cloud offerings according to their priorities and along several dimensions to select whatever is appropriate to their needs. However, it is a difficult task to perform an unbiased comparison and evaluation of all services. Several challenges must be addressed to develop an evaluation model that precisely measures the service level of each cloud provider. This study aims to provide a comparable service analysis for the cloud user to choose among desired services [30].

### 3.2. Compliance

Service provisioning should comply with appropriate policies. The assurance of service compliance comes from the service providers [31]. The CSP assures the customer of their compliance policies such as data protection, data confidentiality, and necessary data security by complying with the international compliance authority. NIST, ENISA, HIPAA, ISO 27001, and CSA are several compliance authorities who provide guidelines to establish the current cloud compliance security standards for the industry. In the Abbreviations Section, the details of the acronyms are presented.

### 3.3. Prediction

Prediction is important in cloud service provisioning. A service user should be ensured of the elasticity and scalability of the services, even during peak hours or when the user suddenly makes an unusually high demand on the resources [32]. In this situation, one of the objectives of the service provisioning selection is that the request should be instantly fulfilled by the service provider. Therefore, the user should be assured of the available required resources on demand with the predictable elastic and scalable services.

### 3.4. Rank

Selecting the best and most appropriate service is a vital factor for the cloud service user. Selecting services depends on comparing and ranking them suitably [31]. A reasonable and acceptable ranking system helps the cloud customer to make decisions about service selection. Therefore, the cloud service ranking system is an important aspect of a fair cloud service comparison and selection process. However, there is a lack of comparison of services across providers due to a lack of common comparable criteria or attributes.

## 4. Major Services of Service Provisioning 

In cloud computing, in the perspective of resource allocation and service provisioning, the services layers are divided into several working layers [33]. There are then four service layers: the application layer (SaaS), the platform layer (PaaS), the infrastructure layer (IaaS), and security as a service (SecaaS) layer. For the list of acronyms, please refer to the Abbreviations Section. Each of these layers provides a specific service for users, which are explained as follows [34].

### 4.1. Infrastructure as a Service (IaaS)

Infrastructure as a service, defined as providers who offer computing and storage resource capacity via vitalization, allowing physical resources to be assigned and split dynamically. A typical application could be an on-line alternative to a word processor or spreadsheet. Several types of virtualization occur in this layer. Along with other resources, it includes computing, network, hardware, and storage. At the bottom layer of the framework, infrastructure devices and hardware are virtualised and provided as a service to users to install the operating system (OS) and to operate software applications. Therefore, this layer is called infrastructure as a service (IaaS). The Elastic Computing Cloud of Amazon (Amazon EC2) and storage by both Elastic Book Store (EBS) and Simple Storage Services (S3) are typical services of this layer.

### 4.2. Platform as a Service (PaaS)

Platform as a service, defined as a provider that offers an additional layer of abstraction above the virtualised infrastructure. The provided software platform trades off restrictions on the type of software that can be deployed for built-in scalability PaaS including mobile operating systems such as Android, iPhone, Symbian, and other OSs, as well as database management and IMS. This layer contains the environment for distributing storage, parallel programming design, the management system for organising distributed file systems, and other system management tools for cloud computing. Program developers are the primary clients of this platform layer. Entire platform resources such as program testing, running, maintaining, and debugging are delivered by the platform directly from this layer. Hence, this form of services in the platform layer is termed platform as a service (PaaS). Classic examples of these services include Google App Engine and Microsoft Azure.

### 4.3. Software as a Service (SaaS)

Software as a service, defined as a provider who supplies remotely run software packages to consumers via the Internet on a utility-based pricing model. Analytical, interactive, transaction, and browsing facilities are included in the application layer. SaaS delivers several simple software programs and applications as well as customer interfaces to the end users. Thus, in the application layer, this type of service is called software as a service (SaaS). By using the client software or browser, the user can connect to services from providers via the Internet and pay fees according to the services consumed, in a pay-as-you-go model. Customer relationship management (CRM) from Salesforce is one of the early SaaS applications. Among other services, Google provides online office tools such as documentation, presentations, and spreadsheets, which are all part of SaaS.

### 4.4. Security as a Service (SecaaS)

The agility offered by the on-demand provisioning of computing resources and the ability to align information technology with business demands are valuable; however, clients are also very anxious about the security risks of cloud computing and the cost of direct control over the security of systems. Although vendors have attempted to satisfy this demand for security by offering security services in a cloud platform, the selection process is still completed [35]. These issues have led to the restricted adoption of cloud-based security services, but the future looks bright for SecaaS, with Gartner predicting that cloud-based security service will be more than triple in many segments. To support both cloud customers and cloud providers, CSA has adopted a new research project to provide greater clarity in the area of SecaaS. It refers to the provision of security applications and services from the cloud to cloud-based infrastructure and software or from the cloud to the customers on premise systems. SecaaS will allow enterprises to make use of security services in new ways that would be more costly if provisioned locally.

## 5. Service Provisioning Requirements

There are several types of service provisioning from which we can make need-based selections, as discussed below.

### 5.1. Agility and Availability

Agility is one of the great advantages of cloud computing, which enables an organization to expand and change its resources quickly without much expenditure. Agility in SMI is measured as a rate-of-change metric, showing how quickly new capabilities are integrated into IT as needed by the business. When considering a cloud service's agility, organisations want to understand whether the service is elastic, portable, adaptable, and flexible. A virtualised optical network is proposed as a key to implementing increased agility and flexibility in a cloud computing environment by providing any-to-any connectivity with the appropriate optical bandwidth at the appropriate time. Jinno and Tsukishima [36] proposed a concept for a virtualised optical network (VON), which is achieved through virtualization in the optical domain as a key to implementing increased agility and flexibility in a cloud computing environment by providing any-to-any connectivity with the appropriate optical bandwidth at the appropriate time.

Hirzalla [37] explored their impact on business agility and software development by sharing best practices and lessons learned through an interactive session that offers insights from previous field engagements. More specifically, they addressed how to realize business agility requirements through the potential synergies between SOA and cloud computing. The concept of business agility originates from the manufacturing industry in the 1980s. In contrast to other concepts such as flexibility, agility refers to the ability of a firm to adapt swiftly to changing environments. Thus, business agility can be defined as the ability to sense and respond to opportunities and threats in an efficient, effective, and timely manner. Consequently, agility is crucial for firms, especially in volatile environments, to stay competitive. In this regard, IT plays an important role in sensing and responding capabilities [38]. Peng et al. [39] proposed a framework for resource provisioning in the cloud through network vitalization. It delivers optimized resources, on-demand scalability, and flexible future CSPs based on the concept of an IaaS framework and IP network virtualisation.

### 5.2. Pricing

The SLA resource allocator acts as the interface between the data centre/CSP and external users/brokers. It supports SLA-oriented resource management. After receiving a service request, the service request examiner and admission control mechanism interpret the submitted request in terms of QoS requirements and ensure resource availability. Then, it requests VMs for resources and determines the allocated VMs. The charges for the service request are determined by the pricing mechanism based on submission time, pricing rates, or the availability of resources. The actual usage of resources is tracked by the accounting mechanism. In addition, the VM monitor tracks the availability of VMs, the dispatcher begins the execution, and the service request monitor mechanism tracks the execution progress of the service requests [1].

Rosenthal et al. [40] express the concern that some users have had unpleasant surprises regarding the costs associated with the unexpectedly heavy use of cloud resources and expecting some cloud vendors to offer suitable throttling services. A remarkable increase of cloud computing service offerings has enabled technologies for service composition, by developing efficient pricing models to foster the resource allocation process and evaluate the services used. Weinhardt et al. [41] present a multidimensional procurement auction for composite services: a model for service value networks based on a graph structure where a path is allocated by the auction mechanism through a network containing price and configurations of the offerings.

Saure et al. [42] proposed a reservation system with finite computing resources over an infinite horizon, where a set of incumbent users submit reservation requests for computing resources ahead of time. The multinomial logit (MNL) framework is used to model customer substitution behavior by adjusting the resource prices in tokens per unit of time and per computing resource. A class of pricing policies called time-of-use (ToU) is considered, and a simple and intuitive algorithm is proposed to formulate the problem. The evaluation showed that the optimal ToU policy outperforms single pricing strategies for customer satisfaction by 3–8%, on average. Teng and Magoulès [43] propose a new resource pricing and allocation policy where users can predict the future resource price while satisfying budget and deadline constraints. By using game theory, the resource price gradually converges to an equilibrium state based on dynamic games where the users can receive a Nash equilibrium allocation proportion without the other competitors' bidding information. The experiments were implemented in a CloudSim simulation.

In cloud computing, resources are provisioned by reservation and on-demand plans. However, a reservation plan is cheaper than an on-demand plan. Due to the uncertainty of the customer's future demands, it is difficult to minimize costs. To address this problem, Chaisiri et al. [44] proposed an optimal cloud resource provisioning (OCRP) algorithm using a stochastic programming model. The OCRP algorithm provisions the computing resources for multiple provisioning stages based on the demand and price uncertainty. By using a deterministic equivalent formulation, sample-average approximation, and Benders' decomposition, the OCRP algorithm can successfully minimize the total cost of resource provisioning in cloud computing environments. Samimi and Patel [45] presented a comparative review of grid and cloud computing economic and pricing models using tariffs and charging. They considered several factors, such as regulations, tax laws, service level agreements, and return on investments, and reviewed the latest economic and pricing models for grid and cloud computing.

The current pricing scheme for cloud computing has significant constraints. Spot instance, the first deployed auction-style pricing model of Amazon EC2, cannot handle untruthful bidding in resource allocation. Hence, [46] addressed the problem of cloud resource pricing by proposing a suite of computationally efficient and truthful auction-style pricing mechanisms. The proposed algorithms can increase revenue by achieving truthfulness without collusion or (*t*; *p*)-truthfulness tolerating a collusion group of size *t* with a probability of at least *p*. Sharma et al. [47] designed and simulated a cloud resources pricing model using financial option theory where the cloud resources are treated as real assets. Then, using the financial model, the cloud resources are priced. The compounded Moore's law is applied to handle the pricing of resources at the required QoS, which can then manage a realistic cloud pricing problem. Recently, cloud computing has emerged as a model in support of “everything-as-a-service.” Motivated by this idea, [48] proposed a personalised pricing strategy for cloud computing, which is defined as pricing as a service (PraaS). This method efficiently meets the demands of the customer and simultaneously maximises the revenue of the providers. The experimental results show that this pricing method is appropriate for the on-demand use of cloud resources and guarantees more revenue for the cloud providers. Li et al. [49] combined the computing and resource swarm algorithms to evaluate the resource price adjustment by the cloud bank model. The pricing strategy includes two important models: the initial price model (IPM) and the resource swarm algorithm price adjustment model (RSAPAM). Hence, resources will reach the most reasonable price over time. Ren and van der Schaar [50] proposed a joint optimization of scheduling and pricing decisions for delay-tolerant batch maximize to maximize the service provider's long-term profit. Dynamic setting and provably efficient dynamic scheduling and pricing (Dyn-SP) algorithms are developed without the necessity of predicting future information. The results indicate higher average revenue with the same average queuing delay.

### 5.3. Security and Trust

Research interest has therefore arisen in developing software engineering techniques to support systems based on the cloud, to enable software engineers to identify security and privacy requirements and to select a suitable cloud service provider based on such requirements [51]. Mouratidis et al. [52] propose a novel framework to support the elicitation of security and privacy requirements and the selection of a cloud service provider. It incorporates a modelling language and provides a structured process that supports elicitation of security and privacy requirements and the selection of a cloud provider based on the service provider's satisfaction of the related security and privacy concerns. This framework illuminates the organisational context by identifying goals, actors, tasks, resources, and plans to identify and analyse the privacy constraints, security and privacy goals, threats, and vulnerabilities relevant to a cloud based system.

Dasgupta and Rahman [53] present a cloud security insurance framework to estimate the coverage of different cloud services. For cloud security insurance, security coverage is important and relevant, as the cost of deploying special protection, detection, and response tools varies and requires well-organised coverage estimation. Hence, an intelligent search and optimization technique for determining the appropriate combination of tools to provide multilevel defence for cloud services is necessary. A framework is developed for calculating security coverage focusing on the security techniques and solution tools of different cloud services. A Java software program named MEGHAND is developed to implement and test this insurance security model and tested for different cloud security models. This framework reports the cloud security coverage problem for risk analysis and for developing insurance models for the cloud. This coverage estimator (MEGHNAD) is primarily useful for cloud insurance providers in meeting customer security expectations while satisfying all service requirements. The cloud provider, however, must use the recommended security products from a multivendor and mix them seamlessly for the desired security coverage. Rigorous risk assessments are needed to ensure the map of policies and procedures. The genetic algorithm optimizing method used in MEGHNAD is scalable to accommodate tool-specific configuration settings, required standards, and compliances for a fine-grained coverage estimate. Arias-Cabarcos et al. [54] introduce a federated identity management (FIM), identified by researchers and experts as a vital security enabler for implantation of cloud computing. However, the current frameworks are limited by the complexity of the dynamic federation between interclouds. A key requirement is trust management to foster collaboration and analyse the FIM process. A set of new metrics is defined to allow the novel form of risk measurements. A generic hierarchical risk aggregation system and cloud-based service provisioning are mentioned as contributions. A set of risk metrics is derived that can serve as an aggregation model for risk calculation. The AHP method is used for independent analysis.

One critical problem is the location of major problems in complex cloud application scenarios; therefore, [55] propose a ranking-based framework named FTCloud for building fault-tolerant cloud applications. Two ranking algorithms are proposed to identify the significant components within the enormous amount of cloud data. Then, they find the best suitable fault-tolerance strategy for each individual component. FTCloud consists of two parts: ranking and optimal fault tolerance selection. A component graph is prepared for component invocations, which then pass through component ranking, where the ranking algorithms are employed. Based on the ranking results, the important components are identified, and the most suitable fault-tolerance strategy is selected. Finally, the ranking of the selected fault-tolerance components returns to the system designer to build authenticated cloud applications. However, the software component graphs used here are limited [56].

To assess the security risks related to cloud computing platforms, a quantitative risk and impact assessment framework (QUIRC) is presented by Saripalli and Walters [57]. It defines risk as a combination of the probability of a security threat event and its severity, measured as its impact. Here, six key security objectives (SO) are identified for cloud platforms, and the typical attack vectors and events are mapped into these six categories. For assessing security risks, the Wideband Delphi method is proposed to collect the information. The advantage of QUIRC is that it offers fully quantitative and iterative convergence for the dependable comparative assessment of the relative robustness of different cloud provider offerings and approaches. A quantitative framework is presented for analyzing and assessing the risks and impacts to the security of cloud-based software deployments, and the advantages of the approach are elucidated. They introduce methods of risk assessment based on probability and impact and the QUIRC framework. Traditional threat modelling can be related to the QUIRC computations via the identification of threat events. To build more reliable input data for QUIRC analysis based on industry verticals and expert knowledge, a Wideband Delphi method is proposed. A quantitative approach provides vendors, customers, and regulatory work groups with the ability to assess the relative robustness of different cloud vendor offerings and approaches in a defensible manner. Moreover, it helps to alleviate the fear, ambiguity, and uncertainty related to cloud platform security issues by ensuring they will be handled effectively. However, its limitation is that it requires the careful collection of input data for probabilities of events, which requires collective industry SME inputs.

Ouedraogo and Mouratidis [58] highlight the importance of an informed choice of CSP to reduce the exposure to insecurity in a cloud context. A well-defined approach called C.A.RE (complete-auditable-reportable) is proposed, which helps to determine CSP security by assessing its completeness and possible risks and vulnerability for the cloud service customer (CSC). Hence, reliability and availability are two important aspects from a user perspective. DoS, natural disasters, and equipment outages are potential threats against cloud services availability and reliability. The assurance of security is defined as when an entity meets the objective of security. The underpinnings of the C.A.RE approach the need of mutual auditability and multiparty trust consideration. The “complete” phase: this phase concerns accumulating the necessary evidence to comply with and support the alignment between the policies implemented by the CSP and the security requirements of the CSC. These security needs and compliance include regulations, laws, security policies, standards, and best practices. Here, a certification is treated as a badge of security assurance for the CSP, for example, ISO/IEC 270001 certification for a data centre security certificate. The completeness is practically determined by considering the set of security requirements identified by the nature of the relevant CSC application. Then, the security mechanism for a certain provider is mapped and analysed to determine to what extent the security requirements are met. The “auditable” phase and its dependent metrics: this phase supplements the completeness metric. The necessary components of the audit are the coverage, depth, rigor, and independence of the verification. The “reportable” phase: according to the scope of the SLA, the CSP conveys security issues or information in a timely manner to the CSC. The completeness indicates the suitability of the CSP's security concerns to the CSC. The second feature ensures the auditability of the security deployed by the CSP. Finally, reporting provides the security transparency information to the CSC. We present the cloud service requirements in Table 1.

### 5.4. Quality of Service

As cloud computing is gaining much attention in the last few years, the idea of IT services through Internet on-demand and pay-as-you-go model also changes. Thus, the exponential escalation of such service and selecting the optimal service provider based on quality of service (QoS) become vital. Salama et al. [59] propose the idea of integrated QoS assured utility model to address the problem of cloud service provider selection based on a multidimensional QoS approach, for satisfying the best utilization of consumer requirement. The proposed mathematical model assists decision makers in selecting the optimal cloud service provisioning, incorporating customer's minimum needs, quality of service, and business profitability and performance criteria. Bao and Dou [60] use finite state machine (FSM) to recommend the legal invocation orders of these services and an improved tree-pruning-based algorithm is proposed for creating the web service composition tree (WSCT) for optimal service selection. First, an improved tree-pruning-based algorithm is introduced to construct the composition tree and then the SAW technique is adopted for service selection.

Zhao et al. [61] address four main issues to solve the service selection problems from multiple service providers such as scalability, flexibility, multiple QoS facility, and automatic user preference support. Therefore, service providers should have the high degree of credibility and service quality. Hence, the trust degree implies the reliability or availability level. Again, Zheng et al. [62] introduce a personalized QoS ranking prediction system framework, CloudRank, which requires no extra service invocations for QoS ranking in cloud services. The past users' experiences are exploited for ranking based approach and accumulate and identify the preferences between a pair of services to obtain a ranking service. Chan and Chieu [63] propose a mechanism by evaluating specific performance and QoS attributes based on singular value decomposition (SVD) to select the best service provider for a user application with a set of predefined requirements. A user sends a request for the requirement to execute the application into the cloud service provider mapper which dynamically provides the service provider according to application demands.

Li et al. [64] describe a method to achieve optimization in clouds by supporting the developers to enable runtime optimization employing an optimization algorithm. It maximizes profits in the cloud constrained by QoS and SLAs within the large variety of workloads. Srivastava and Sorenson [65] propose a technique that overcomes the restriction and compares functionally equivalent services on the basis of the customers' perception of the QoS attributes rather than the actual attribute values. Goscinski and Brock [66] propose a framework which provides the application of the resources via web services framework (RVWS) to offer higher level abstraction of clouds in the form of a new technology to service provisioning resource publication, discovery, and selection based on dynamic attributes. Automatic service publishing, selection, and discovery of required services are still one of the research priorities. In addition, this model explores the ways of publication, discovery, selection, and use of cloud resources as services without specialized knowledge by using dynamic and current attributes through web service WSDL documents to help, discover, and select essential services and resources based on user requirements.

## 6. Metrics

A set of business-relevant key performance indicators (KPIs) that provide a standardised method to measure the business services are needed. The SMI framework provides a holistic view of the QoS needed by the customers for selecting a CSP based on accountability, agility, assurance of service, cost, performance, security and privacy, and usability. There are currently few publicly available metrics that define these KPIs and compare cloud providers. SMI is the first effort in this direction. The defined high-level attributes are accountability, agility, cost, performance, assurance, security and privacy, and usability. The proposed metrics for cloud KPI are service response time, sustainability, DCiE and PUE (the most prominent metrics used to measure the energy efficiency of a cloud computing service), suitability, accuracy, interoperability, availability, stability, adaptability, usability, throughput and efficiency, and scalability [18].

Li et al. [67] proposed a metrics catalogue with multifold usability. This catalogue can be used as a dictionary to conveniently look up suitable metrics for cloud deployment. The existing evaluation metrics in the catalogue can help in developing metrics for research. Moreover, the preliminary metrics may help to better implement the evaluation of cloud services. Li et al. [68] developed a set of tools to measure the metrics under four major providers: AWS, AppEngine, Azure, and cloud servers. The devised tool is simple and can be easily extended to other cloud platforms to measure application performance. In addition, Bojanova and Samba [69] propose to determine the relative efficiencies of the different cloud computing models by measuring and analyzing the following cloud computing infrastructure metrics: hardware costs, software costs, and real-time provisioning costs. Real-time provisioning is integrated into the service management system by applying automated tools. Maiya et al. [70] introduce cloud manageability metrics by defining the user's role, major use cases that the user performs, and metrics. Different platforms are used to validate this strategy. The proposed metrics are interfaces, documentation, time to learn, number of steps, time taken, and ease of use. Finally, [71] propose the most relevant metrics and figures of merit for the evaluation of customer cloud benchmarks. These metrics are provisioning latency, provisioning throughput, and runtime performance, which are measured by latency, throughput, and bandwidth. The aforementioned metrics can be compared to the following attributes: scalability, stability, and reliability.

## 7. Models, Methods, and Techniques of Service Provisioning

The used methods, models, and techniques for successful and efficient provisioning of services have a paramount importance. For these discussions, we use several acronyms and please refer to the Abbreviations Section. Hence, we discuss the criteria of service provisioning as follows.

### 7.1. Service Provisioning Models

The service measuring index (SMI) is a service measurement model based on business model of the International Standard Organization (ISO). The SMICloud model is proposed by Garg et al. [18] which lets users compare different cloud offerings, according to their priorities and along several dimensions, and select whatever is appropriate to their needs. Several challenges are tackled in realizing the model for evaluating QoS and ranking cloud providers. The SMICloud systematically measures all the QoS features proposed by cloud service measurement index consortium (CSMIC) and ranks the cloud services based on these business services. Again, Li et al. [68] introduce CloudCmp, a systematic cloud service performance and cost comparator system. It assesses the elastic computing, storage, and networking services promised by cloud metrics, which have an impact on performance of customer applications. CloudCmp safeguards the fairness, participation, and compliance of these assessments, compromising limiting measurement cost. By using CloudCmp, most of the cloud customers today can find offered services from cloud service provider very widely in terms of efficiency and price and calculate the need for a thoughtful provider selection. Han et al. [72] present the cloud service selection framework which employed a recommender system (RS) that selects the best services from different cloud providers according to the customer needs. The Cloud Provider (CP) registers their services in the web portal through CSRS system and users put their requirements and get recommendation by the web portal. If a CP wants to register, the request is passed to the request manager through the web portal after the evaluation process and then sends to resource register after calculating the S-rank and QoS values of CPs and storing in resource repository.

### 7.2. Brokerage Aided Provisioning

Sundareswaran et al. [73] describe the problems of finding the best price and services are addressed from the huge pool of services from the cloud service providers. A novel brokerage based architecture is proposed to use a unique indexing technique for efficient service selection by employing algorithms which aggregate and select the optimal option. In this model, the cloud broker collects the service provider's properties and similar properties are analyzed and indexed by the cloud broker and using it when receiving request from customer for the best matched service. Le Duy et al. [74] introduce a new benchmark to evaluate and compare cloud brokers. Cloud broker challenge (CBC) explains the cloud providers, cloud consumers, and goals with five variety levels of complexities. CBC benchmark is useful for evaluation and comparison of unbiased brokers and feasible for real-life cloud brokers. The design and development of software agents for cloud service discovery, service negotiation, and service composition play an important role. Sim [75] introduces an agent-based paradigm for constructing software tools and test beds for cloud resource management. He develops Cloudle—an agent-based search engine for cloud service discovery to show effective agent-based negotiation mechanisms and agent-based cooperative problem solving techniques effectively adopted in automatic cloud service composition. Moreover, agent-based problem solving techniques such as acquaintance networks and the contract net protocol are employed by Gutierrez-Garcia and Sim [76] for creating a self-organizing service composition framework. In this model, cloud service composition framework provides generic agent behaviours to handle ad hoc web service workflow specifications. Finally, self-organizing service composition is supported by contracts among cloud participants which mapped to service level agreements in cloud computing environments. Amato et al. [77] in the mOSAIC project design knowledge based representing resources and domain concepts of semantic web ontologies and rule based support tool, the semantic engine. It aids the user to abstract the requirements in a vendor independent way to compare the different offers of providers with the user proposal and retrieves the best offer.

### 7.3. Policy Ensured SLA

In cloud computing, contracts between users and traders are recognized as service level agreements (SLAs), mentioning the terms and conditions of service usage. Service level agreements are established between service consumers and providers and define a number of obligations and rights for both sides. However, the increasing number of service offerings is so rapid and there is a lack of a standard for specification; manual service selection is an expensive task, averting the successful implementation of on demand ubiquitous computing. Therefore, automatic methods for matching SLAs are essential. Redl et al. [78] propose a method to select semantically equal SLA elements from differing SLAs by employing several machine learning algorithms. In addition, this method enables autoselection of optimal service offerings for cloud services. A framework is presented to automatic SLA management by a simulation-based study to establish several significant advantages of this approach for cloud customers. Research on SLA management focuses on SLAs with rights for consumers and obligations for providers, keeping the two parties balanced interest. Spillner and Schill [79] present a solution of monitoring data at runtime and feeding it back into the service registry to adjust descriptions and make contract template derivation as a more realistic process. Emeakaroha et al. [80] introduce detection SLA violation infrastructure (DeSVi), the novel architecture for monitoring and detecting SLA violations in cloud computing infrastructures. The main components of the architecture are the automatic VM deployer, responsible for the allocation of resources and for mapping of tasks, application deployer, responsible for the execution of user applications, and LoM2HiS framework, which monitors the execution of the applications and translates low-level metrics into high-level SLAs. However, this proposed system is capable of monitoring only a single cloud data center. On the one hand, SLA violation should be prevented to avoid costly penalties and on the other hand providers have to efficiently utilize resources to minimize cost for the service provisioning. Few approaches are limited to simple workflows and single task applications. Bouchenak [81] introduces a systematic and synchronized integration by the definition of SLA aware cloud and explains the automated cloud control for building SLA aware dynamic elastic clouds. Finally, Chi et al. [82] describe a framework by employing novel data structure. Pearson and Sander [83] introduce a policy based mechanism of service provider assessing the risk-based semiautomated system which drastically reduces the transaction to lower the cost of selecting desired CSP. This ensures compliance and trustworthiness of service providers.

### 7.4. Heuristic and Holistic Perspective

Song et al. [84] introduce a framework for task selection and allocation to enhance resource utilization for PCP by exploiting an adaptive filter. To optimize the goal of a heuristic algorithm for optimizing the tradeoff between QoS of the tasks and utilization of resources a tradeoff metric is introduced. A VM-based overall resource structure for computing resource utilization is presented. Simulation study shows that algorithm performs better than other existing algorithms [85, 86]. Moreover, Beloglazov et al. [87] define an architectural framework and principles for energy aware heuristics provision data center resources to client applications in cloud computing. Energy efficient resource allocation policies and scheduling algorithms are introduced by considering QoS expectations and power usage characteristics. The approach is validated by a performance evaluation study using the CloudSim toolkit. Again, Casalicchio and Silvestri [88] propose autonomic service provisioning and resource management of cloud-based systems especially for IaaS providers. Thus, this system has four alternatives implementation which has different degree of control on the various components of the autonomic cycle.

Ferrer et al. [30] incorporate the service provider (SP) and infrastructure provider (IP) with toolkit which optimize the whole service life cycle. Each core component of the toolkit provides common services, which are needed for service deployment and execution. Again, the criteria followed by IP are past performance and legal and security aspects. The calculation is conducted by considering a value for each criterion from 0 to 1. Finally, the assessment is achieved by implementing by Dempster Shafey analytical hierarchy process (DS-AHP) [89] as the service provisioning system is important in the user's perspective of cloud service performance. Hence, for network virtualization it is a vital attribute of next-generation Internet-based service provisioning approach to integrating networking and cloud computing. Duan [90] introduces a holistic approach of the application of the SOA in network virtualization for composing network and cloud services and studied modelling and performance analysis on network virtualization for composite network-cloud service provisioning.

### 7.5. Cloud Service Provisioning Based on MCDM

Multicriteria decision making (MCDM) is a well-established area in the field of operations research and has proven its effectiveness in addressing different complex real-world decision-making problems. Rehman et al. [91] present a comparative case study involving infrastructure as a service cloud and use MCDM techniques to select the best service on the basis of actual performance measurements by a third party monitoring service against five different criteria. We present a comparative study of service provisioning techniques in Table 2.

Analytical hierarchy process (AHP) is a tool for decision makers to be able to do more informed decisions regarding their investment in such technologies. The AHP is a multiobjective, multicriteria decision-making approach, which employs a pair-wise comparison procedure to arrive at a scale of preferences among a set of alternatives. AHP enhances the decision making towards transform subjective judgments into objective measures [100]. In AHP, an input is asked to give ratios for each pairwise comparison between issues for each criterion in a hierarchy and also between the criteria [101]. The pairwise comparison results are displayed in a hierarchy with a weight for each criterion, providing both qualitative as well as quantitative characteristics here. In addition, the technique is for order preference by similarity (OPS) to an ideal solution (TOPSIS) to aid service consumers and providers for analyzing available services with fuzzy opinions. Fuzzy TOPSIS methods are now popular in dealing with imprecise information.

### 7.6. Algorithmic Techniques

In the area of multiobject optimization, genetic algorithm is a famous search method. Genetic algorithm based search is employed to find out the best service variant among the current context by Vanrompay et al. [102]. Then, the choice services are deployed in an optimal way which serves the demands of the service running on mobile systems. Their model has several requirements such as deployment of an intelligent artificial learning mechanism, scalable variants, resource constraints of mobile devices, bandwidth limitation, and run time QoS properties that should be considered. Zhao et al. [103] propose an optimized task scheduling algorithm based on genetic algorithm to schedule independent and divisible tasks to adapt to different computation and memory requirements. The algorithm is in the heterogeneous system, and dynamic scheduling is also considered and accordingly GA is designed to solve combinational optimization problem. Again, Dutta and Joshi [95] propose a genetic algorithm based approach to cost based multi-QoS job scheduling model in cloud computing. It guarantees the best solution in finite time. A genetic algorithm has been developed to provide a better scheduling in a cloud environment. Analysis and a number of results show that it ensures a good profit for the different cloud providers. The real execution time of job in different system as well as soft deadline and penalty cost in the algorithm is also considered.

Wei et al. [92] present a game-theoretic method for scheduling cloud-based computing services with collaborative QoS requirements. Game theory is employed to solve the problem of resource allocation. A binary integer programming method is proposed to solve the independent optimization, and an evolutionary mechanism is designed to minimize their efficiency losses. The algorithms consider both optimization and fairness into account which finally reveal that Nash equilibrium always exists for game which has feasible solutions for resource allocation. Again, Rao et al. [93] propose a game-theoretic approach for the provisioning and operation of the infrastructure by considering qualitative effects of cost and strategy model. The Nash equilibrium under different formulations computes in polynomial time and derives provisioning choices to ensure the capacity C with probability PS. In addition, Ardagna et al. [94] introduce a game theory based model for the run time management in service provisioning problem especially for IaaS provider capacity among multiple competing SaaS.

## 8. Research Issues and Challenges

This section presents several important open issues and research challenges as well as research directions for successful service provisioning deployment and implementation.

Service availability becomes more important in a dynamic environment. Applications require intensive interaction between the end-user and the cloud service. Hence, service disruption, network congestion, poor signal, and node failure are highly undesirable in service provisioning. When a node moves frequently within the network or changes its point of attachment, many mobile cloud applications demand optimal service through the most suitable node. Again, the scalability of services is a challenging aspect of distributed application processing in cloud computing. Remote application processing is deficient in the centralised management of the distributed platform. A challenging issue in local distributed application frameworks (APFs) is the unavailability of centralised resources. When a remote service provider is unavailable, remote services become inaccessible, which hinders the objectives of availability of services in a distributed computing paradigm. In addition, the QoS requirements are also evolving with the evolution of the cloud, and therefore service provisioning requires highly reliable good service quality. Similar services and functionalities are provided by the different CSPs, which makes it difficult for customers to select the best and most appropriate service. Optimal provider selection based on predetermined quality of service (QoS) requirements becomes vital. Moreover, the users' past experiences are exploited in a ranking-based approach to accumulate and identify the preferences between pairs of services to obtain a service ranking. Thus, this approach gets benefits from past user experience. However, user response does not always reflect true feedback. It is necessary to adjust and justify the feedback using statistical techniques to make it as error-free as possible. Furthermore, SLAs are legally binding for both parties, recognised as the terms and conditions of using the service. Because of the fast-growing number of promising service offers and the lack of a standard specification of services, manual service selection is an expensive task, preventing the successful implementation of ubiquitous computing on demand. Therefore, automatic methods for matching SLAs are essential. Finally, several mathematical and statistical methods and model-based issues are proposed and validated. Service provisioning requires a precise and efficient artificial intelligence learning mechanism, and in the planning stage the number of variants, resource constraints, bandwidth limitations, and properties should of course be considered with respect to run time, hardware, and software.

## 9. Conclusion

Cloud computing is currently an emerging paradigm that envisions a new paradigm of “everything-as-a-service,” hence, virtualizes physical resources, infrastructure, and applications which are being provided through service provisioning in the cloud. The growing adoption of cloud services suggests clear and distinct promises within the cloud industry. Due to the increasing number of cloud providers and the variety of service offerings, it has become difficult for new customers to choose the best provisioned services. Therefore, we have clearly identified service provisioning techniques, mechanisms, and several approaches that must be understood to evaluate the provisioned services in terms of user requirements and costs. Hence, continuous service provisioning that satisfies the user requirements is a mandatory feature for the cloud user and vitally important in cloud computing service offerings. Therefore, we reviewed the state-of-the-art service provisioning objectives, essential services, topologies, user requirements, necessary metrics, and pricing mechanisms. In addition, we synthesized and summarized different provisioned techniques, approaches, and models through a comprehensive literature review. Moreover, a proposal of the thematic taxonomy of cloud service provisioning is presented. Finally, open research issues are categorized and identified for future research directions.

## Figures and Tables

**Table 1:** Requirements of cloud service provisioning requirements.

Requirements	Features	Solving approaches	Attributes	References
Agility and availability	Virtualised optical network (VON)	Appropriate optical bandwidth at the appropriate time	Any-to-any connectivity	Jinno and Tsukishima [36]
	Combining SOA and cloud computing.	An interactive session that offers insights from previous field engagements	Realizing business requirements	Hirzalla [37]
	A framework for resource provisioning	Through network virtualisation	Optimised resources, on-demand scalability	Peng et al. [39]

Pricing	Multidimensional procurement auction for composite services	Based on a graph structure	The auction mechanism	Weinhardt et al. [41]
	Single pricing strategies for customer satisfaction	Multinomial logit (MNL) framework is used	Pricing policies called time-of-use (ToU)	Saure et al. [42]
	Resource pricing and allocation policy	Future resource price prediction	Game theory and implemented in CloudSim simulation	Teng and Magoulès [43]
	Using tariffs and charging	Regulations, tax laws, and SLA	Pricing models	Samimi and Patel [45]
	Joint optimisation of scheduling and pricing decisions	Dynamic scheduling and pricing (Dyn-SP) algorithms	Higher revenue with the same queuing delay	Ren and van der Schaar [50]

Security and trust	Secured cloud service selection	Secured service framework	Software engineering	Mouratidis et al. [52]
	Insurance models for cloud security	Cloud security insurance framework	MEGHAND	Dasgupta and Rahman [53]
	Federated identity management	Hierarchical cloud based risk aggregation system based service provisioning	Risk metrics	Arias-Cabarcos et al. [54]
	Ranking based fault tolerant framework	FT cloud model	Ranking with optimal fault tolerance	Zheng et al. [55]
	Quantitative risk and impact management	QURIC framework	Wideband Delphi method	Saripalli and Walters [57]

QoS	QoS-based utility optimum service selection	Optimization with minimum requirements	Response time, availability, and throughput	Salama et al. [59]
	Service selection carried out by CWS tree constriction	FSM, SAW, tree pruning algorithm	Possible execution path	[60]
	System model of personalized user support to optimize QoS support.	Algorithm	Trust, response time, price, and platform	Zhao et al. [61]
	QoS prediction system (CloudRank) considering past user experience	KRCC and ranking prediction algorithm	Preference of services	Zheng et al. [62]

**Table 2:** Comparison of service provisioning techniques.

Service provisioning techniques	Features	Solving approaches	Attributes	References
Algorithmic	A cloud-based computing services scheduling with collaborative QoS requirements	Binary integer programming method	Optimization and fairness	Wei et al. [92]
	Considering qualitative effects of cost and strategy model	Nash equilibrium under different formulations	Capacity and probability	Rao et al. [93]
	Run time management in service provisioning in IaaS	Distributed algorithm	Equilibrium efficiency	Ardagna et al. [94]
	Cost based multi-QoS job scheduling model	Soft deadline and penalty cost	Better scheduling	Dutta and Joshi [95]

MCDM	Task oriented resources allocation	Reciprocal and induced bias matrix	Bandwidth, task costs, and time	Ergu et al. [96]
	A distributed resource management	Considering SLA and QoS	Realizing user needs	Khaddaj [97]
	A dynamic autonomous resource management and scalability	PROMETHEE architecture	Suitable for large data centers	Yazir et al. [98]

SLA and Policy based brokering	For autoselection of SLA from different offerings	Machine learning algorithms	SLA mapping	Redl et al. (2012) [78]
	Knowledge based sources and services in mOSAIC project	Semantic web Ontologies rule based support tool	Requirements and services	Amato et al. [77]
	Compare and evaluate cloud broker by CBC benchmark	Cloud service selection (CSS) algorithm	Query encoding, k-nearest neighbor	Le Duy et al. (2012) [74]
	Best offering selection by brokerage based architecture	Indexing technique B+-tree	Encoded and analyzed, index key	Sundareswaran et al. (2012) [73]
	Monitoring and detecting SLA violation	DeSVi architecture	Low level metrics to high level SLAs	Emeakaroha et al. (2012) [80]
	SLA aware cloud considering by data structure SLA tree	SLaaS, SLA tree	SLA aware provisioning	Bouchenak [81], Chi et al. [82]
	A policy based mechanism of service provider selection	Assessing risk by semiautomated system	Low cost with trust and compliance	Pearson and Sander [83]

Heuristic and holistic	Energy aware heuristics provision of data center resources	Energy efficient allocation policies and algorithms	Power usage, QoS	Beloglazov et al. [87]
	Four architectural schemas for autonomic resource allocation	Four alternative degree of control	Autonomic management	Casalicchio and Silvestri [88]
	Optimized service life cycle for dependable adaptive dynamic service provisioning	Dempster Shafey analytical hierarchy process (DS-AHP)	Past performance, maintenance, security, and legal	Ferrer et al. [30]
	Accessing federated architecture dynamically	Meta brokering concept	Heterogeneous IaaS aggregation	Kertesz et al. [99]

## Abbreviations

AHP: Analytical hierarchical process
CC: Cloud computing
CSA: Cloud security alliance
CSMIC: Cloud service measurement index consortium
ENISA: European Network and Information Security Agency
FSM: Finite state machine
GA: Genetic algorithm
HIPAA: Health insurance portability and accountability act
IaaS: Infrastructure as a service
IVF: Intervalued Fuzzy
MCC: Mobile cloud computing
MCDM: Multicriteria decision making
NIST: National Institute of Standards and Technology
PaaS: Platform as a service
QoS: Quality of services
RVWS: Resources via web services framework
SAW: Simple additive weighting
SecaaS: Security as a service
SLA: Service level agreement
SMI: Service measurement index
SOA: Service oriented architecture
SaaS: Software as a service
TOPSIS: Technique for order preference by similarity (OPS) to an ideal solution (TOPSIS)
VM: Virtual machine
VON: Virtualized optical network.
